# Publisher’s Note: Welcome to *Dermatopathology*—A New Society Journal at MDPI Publishers

**DOI:** 10.3390/dermatopathology7010003

**Published:** 2020-06-30

**Authors:** Peter Roth

**Affiliations:** MDPI, St. Alban-Anlage 66, CH-4005 Basel, Switzerland; roth@mdpi.com

The European Society of Dermatopathology (ESDP) decided to publish their official journal with MDPI Publishers, Basel, Switzerland. ESDP follows the same direction as 113 other societies before who already decided to cooperate with MDPI. This trend fits with the overall growth of our journal program and the dynamic activities of MDPI all over the globe.

What is behind this trend and why are more and more authors and editors submitting their articles or contacting us?

Yes, there are a lot of advantages for the authors in open access publishing—especially with the non-restrictive cc: by 4.0 license. However, being a pioneer and now the largest open access publisher in the world cannot be the reason, because, meanwhile, more and more other publishers are also intensifying their open access activities.

While talking with ESDP during their decision process, we learned that a consequent policy pays off. Our policy to keep all our services under one roof is not always easy to manage but it not only improves the efficiency of our services but it also keeps them on reasonable pricing scales.

STM publishing is a well-controlled market and keeping quality at its best is not just a target—it is a must!

In the name of the new *Dermatopathology* Editorial Office and all our employees, let us express a hearty ‘Welcome to *Dermatopathology*’ and the European Society of Dermatopathology!

This journal is going to appear with four issues on a quarterly basis.

Good luck!

Peter Roth,

Publisher

June 25, 2020

